# Seasonal vitamin D variability and its effects on the innate immune response during human endotoxemia

**DOI:** 10.1186/cc9791

**Published:** 2011-03-11

**Authors:** MJ Van den Berg, M Kox, AJ Van der Ven, JP Wielders, P Pickkers

**Affiliations:** 1 Radboud University Nijmegen Medical Centre, Nijmegen, the Netherlands; 2 Meander Medical Centre, Amesfoort, the Netherlands

## Introduction

In the past several years, an important immuno-modulatory role for vitamin D has been identified. At high latitudes, seasonal vitamin D deficiency is common due to due to low UV-B radiation exposure, which is necessary for the synthesis of vitamin D. In this retrospective study we investigated whether vitamin D levels are subject to seasonal variation and whether plasma levels of vitamin D correlate with the extent of the innate immune response during human endotoxemia.

## Methods

Plasma levels of 25-hydroxyvitamin D3 were determined in samples obtained just prior to administration of an intravenous bolus of 2 ng/kg endotoxin (lipopolysaccharide derived from *Escherichia coli *O:113) in 114 healthy male young volunteers. Plasma levels of the inflammatory cytokines TNFα, IL-6, IL-1RA and IL-10 were determined serially after endotoxin administration. Correlation analysis was performed to investigate the relationship between vitamin D status and inflammatory cytokine levels.

## Results

Vitamin D levels were not subject to seasonal variation in the studied population. Furthermore, vitamin D levels did not correlate with peak cytokine levels or areas under the curve of cytokine time courses. Finally, vitamin-D-deficient subjects (< 40 nmol/l) displayed an identical innate immune response compared with vitamin-D-sufficient subjects.

## Conclusions

Vitamin D levels in young healthy males appear to be stable throughout the year. Plasma levels do not correlate with the extent of the innate immune response during human endotoxemia. These findings question the role of vitamin D in modulation of the innate immune response.

